# Interactive contour delineation and refinement in treatment planning of image‐guided radiation therapy

**DOI:** 10.1120/jacmp.v15i1.4499

**Published:** 2014-01-06

**Authors:** Wu Zhou, Yaoqin Xie

**Affiliations:** ^1^ Key Laboratory for Health Informatics Shenzhen Institutes of Advanced Technology, Chinese Academy of Sciences Shenzhen China

**Keywords:** contour delineation, curve refinement, treatment planning, IGRT

## Abstract

The accurate contour delineation of the target and/or organs at risk (OAR) is essential in treatment planning for image‐guided radiation therapy (IGRT). Although many automatic contour delineation approaches have been proposed, few of them can fulfill the necessities of applications in terms of accuracy and efficiency. Moreover, clinicians would like to analyze the characteristics of regions of interests (ROI) and adjust contours manually during IGRT. Interactive tool for contour delineation is necessary in such cases. In this work, a novel approach of curve fitting for interactive contour delineation is proposed. It allows users to quickly improve contours by a simple mouse click. Initially, a region which contains interesting object is selected in the image, then the program can automatically select important control points from the region boundary, and the method of Hermite cubic curves is used to fit the control points. Hence, the optimized curve can be revised by moving its control points interactively. Meanwhile, several curve fitting methods are presented for the comparison. Finally, in order to improve the accuracy of contour delineation, the process of the curve refinement based on the maximum gradient magnitude is proposed. All the points on the curve are revised automatically towards the positions with maximum gradient magnitude. Experimental results show that Hermite cubic curves and the curve refinement based on the maximum gradient magnitude possess superior performance on the proposed platform in terms of accuracy, robustness, and time calculation. Experimental results of real medical images demonstrate the efficiency, accuracy, and robustness of the proposed process in clinical applications.

PACS number: 87.53.Tf

## I. INTRODUCTION

Image segmentation is typically applied to locate objects and boundaries, and it is also important for feature extraction, image measurements, and object display in medical images. It is the process of delineation structures of interest from images. Generally, the boundary delineation is an initial step before performing high‐level tasks, typically for the purpose of some further processing such as analyzing the characteristics of ROIs or making quantitative measurements to monitor disease progression. Segmentation techniques have been applied to delineate a wide variety of organs from medical images data acquired using a wide range of modalities. In some clinical applications, it may be essential and useful to extract boundaries of ROIs from computerized tomography (CT) images, magnetic resonance (MR) images, or ultrasound images. Additionally, boundary delineation is often required for visualization in surgical planning or guidance.

Radiotherapy is an image‐guided intervention, and imaging is involved in every key step of the process.^(1)^ The evolution of radiation therapy has been strongly correlated with the development of imaging techniques. The term IGRT is employed loosely to refer to newly emerging radiation planning, patient setup, and delivery procedures that integrate cutting‐edge image‐based tumor definition methods, patient positioning devices, and/or radiation delivery guiding tools. These techniques combine new imaging tools, which interface with the radiation delivery system through hardware or software and state‐of‐the‐art 3D conformal radiotherapy (3D CRT) or intensity‐modulated radiation therapy (IMRT), and allow physicians to optimize the accuracy and precision of the radiotherapy by adjusting the radiation beam based on the true position of the target tumor and critical organs. This increased accuracy justifies a smaller clinical target volume to planning target volume (CTV‐PTV) margin, decreasing the consequent collateral damage to normal tissues. While IGRT is certainly a step forward for radiation oncology, the efficacy of these image‐guided treatments depends on a treatment plan optimized using these images.

Perhaps the key issue in image guidance is how the information is used to modify treatment. If the target and organs at risk (OARs) can be delineated on online volumetric images, it is possible to generate a new plan in each treatment day. Replanning theoretically provides the highest precision and does not need specialized hardware such as the robotic couch. However, online replanning requires superior online image quality, as well as fast and robust algorithms to perform automatic ROI delineation, dose calculation, and beamlet weight optimization. The accurate contour delineation of the target and/or organs at risk (OAR) is essential in treatment planning for image‐guided radiation therapy (IGRT).^(1)^

Segmentation or boundary delineation approaches vary a lot in terms of their sophistication and the amount of required user inputs.^(2)^ Generally, segmentation can be performed manually, semiautomatically or fully automatically. Manual techniques allow users to outline structures using software such as the ANALYZE package,^(3)^ and the manual segmentation may be more accurate but time‐consuming and tedious for users. More seriously, it is cause of interobserver variation or bias. Semiautomatic techniques may allow the user to have some control or input into the segmentation process, combined with some automatic process using computer algorithms. Semiautomatic approaches based on thresholding, region growing,^4^, ^5^ and deformable models^6^, ^7^, ^8^, ^9^ are widely considered in numerous applications. Finally, fully automatic techniques require no user input and often make use of some prior knowledge from the anatomy being segmented to produce the segmentation or delineation.^(10)^ Two examples of these approaches are atlas‐based segmentation^(11)^ and statistical shape models.^(12)^ Due to laborious nature of manual segmentation and the pressures on clinicians’ time, segmentation or delineation is still a very active research field, and being able to design an optimal segmentation approach that fulfills the necessities of clinical applications is extremely essential for medical clinicians.

Numerous techniques/methods had been proposed for automatic segmentation in IGRT,^13^, ^14^, ^15^ and these methods are generally based on deformable models or atlas for specific organs (e.g., liver). Campadelli et al.^(16)^ reported a review study about the semiautomatic and automatic liver segmentation techniques, and then described a fully automated method based on gray level. There is a growing interest in the use of autocontouring software for radiation therapy.^17^, ^18^, ^19^, ^20^, ^21^, ^22^, ^23^, ^24^ Atlas‐based segmentation has been shown to be very efficient to automatically delineate critical structures.^(19)^ Isambert et al.^(20)^ concluded that an atlas‐based autosegmentation tool was robust and reliable for automatic delineation of large structures in the brain; however, manual contouring was still needed for smaller structures. Huyskens et al.^(21)^ validated the use of an autosegmentation tool for prostate cancer patients based on CT datasets. The accuracy of the automatically contoured prostate was scored as ‘good', indicating that only minor manual adjustments were needed. For the autocontoured bladder they reported a score between ‘excellent’ and ‘good'. La Macchia et al.^(22)^ systematically evaluated three commercial software solutions (VelocityAI 2.6.2, Velocity Medical Solutions, Atlanta, GA; MIM 5.1.1 by MIMVista, Cleveland, OH; and ABAS 2.0 by CMS‐Elekta, Stockholm, Sweden) for atlas‐based segmentation for adaptive therapy in head and neck, prostate, and pleural cancer, and all the ROIs obtained with automatic contouring (AC) were successively corrected manually. A clinical validation study on atlas‐based autosegmentation for head and‐neck cancer patients was recently published by Teguh et al.^(23)^ They showed that geometric differences between autocontours generated with ABAS and corresponding edited contours were relatively small. Dice coefficients of 0.8 for the CTV neck levels and 0.9 for the salivary glands, and mean contour distances of 1.5 and 1.1 mm, respectively, were reported. This study also showed that by editing autocontours, the time needed for delineation compared to manual contouring was significantly reduced from 180 to 66 min on average. Consequently, Voet et al.^(24)^ proposed that editing of CTV neck contours generated by ABAS was essential to avoid large (local) underdosages in the target volume.

There are also toolkits provided by popular commercial software for treatment planning in IGRT, such as the contour tools provided by iPlan, Eclipse, and Pinnacle. The iPlan RT image (BrainLAB, Feldkirchen, Germany) provides automatic target delineation for structures, and fast contouring is achieved with unique, atlas‐based automatic organ segmentation. So it is a fully atlas‐based automatic method. In Eclipse treatment planning system (Varian Medical Systems, Palo Alto, CA), SmartAdapt contouring is achieved by automatically deforming and propagating initial contour to match the current anatomy, and editing or fine tuning the changes using a variety of 2D and 3D contour edition features. Thus it is a semiautomatic method that combines automatic contouring and manual revision. Pinnacle (Philips Healthcare, Andover, MA) is developed for radiation therapy planning system. Contours are completed on Pinnacle using the autocontour and autothreshold tools available. The model based segmentation (MBS) software of Pinnacle includes an anatomic library of 3D patient organ structure models, which reduces the time oncologists spend manually drawing contours. Therefore, it is also an automatic atlas‐based method for contour delineation.

Although a lot of automatic or semiautomatic contour delineation approaches have been proposed, few of them can fulfill the necessities of applications in terms of accuracy and efficiency. Both thresholding and region growing methods are relatively straightforward for automatic segmentation, and they work only using the intensities in images and do not impose constraint on the shape of resulting delineation objects. In addition, deformable models, such as active contour models or Snakes,^7^, ^8^ can move and deform the initial delineation according to an energy term. However, the optimization of parameters in minimizing the energy is generally slow for applications, due to iteratively adjustment of the contour to energy minimization. One contour detection method used edge‐following algorithm based on intensity gradient and texture gradient features proposed for medical images.^(25)^ To the best of our knowledge, no automatic method for delineation is perfect and no generic automatic algorithm has been presented to work properly.^(2)^ Therefore, development of accurate and fast contour delineation is necessary for this challenging task.

Meanwhile, clinicians may like to analyze the characteristics of ROIs and adjust contours manually for the purpose of making quantitative measurements in the delineation process. To this end, semiautomatic methods consider the case of both manual and automatic contour delineation, and interactive tool for contour delineation is required for such cases. The designed tool should allow users to freely delineate ROIs in medical images in a very efficient and highspeed manner. In addition, the initially manual delineation should be refined accurately and fast in order to eliminate drawbacks of manual delineation, which is prone to observer error or bias. The objective of this work is to develop an efficient tool for interactive contour delineation in medical images that can be directly used in the case of IGRT. The proposed approach to segmentation or contour delineation will be a semiautomatic technique, which preserves the advantages of fully automatic techniques using computer algorithms and fulfills clinical necessities of manual techniques.

Interactive tools have been proposed for segmentation or contour delineation before,^26^, ^27^, ^28^, ^29^, ^30^, ^31^ and those methods are mostly related to our work. Barrett and Mortensen^(26)^ proposed a semiautomatic boundary extraction method, in which user can click on a point on the boundary of a region and drag the cursor roughly around the region, an automatic process will then find the best path according to a predefined criterion from the start point to the current cursor. Contour delineation is achieved by tracing the boundary by hand, and this method was used to segment medical images from CT, MRI, and ultrasound. Jackowski et al.^(27)^ developed an interactive tool for 2D or 3D segmentation based on parametric curve and surfaces. It firstly described an automatic iterative thresholding method to obtain coarse segmentation results, and then by moving control points for a RaG curve or a RaG surface interactively, the segmentation results were revised. De Bruijne et al.^(29)^ developed an interactive method for segmentation of abdominal aortic aneurysms. After manual segmentation of the first slice, the method automatically detects the contour in subsequent slices using the results from the previous slices as a reference. If an obtained contour is not correct, the user can intervene and provide an additional manual reference contour using a fitting procedure. Recently, Hu et al.^(30)^ proposed an approach of interactive semiautomatic contour delineation using statistic conditional random fields framework to reduce the time and effort required of expert users. After an initial segmentation on CT slice, the user marks the target organ and nontarget pixels with simple brush strokes. The approach automatically calculates statistics from this information to determine the parameters of an energy function containing both boundary and regional components. The method uses a conditional random field graphical model to define the energy function to be minimized to obtain an estimated optimal segmentation. Liver and kidney segmentations in CT images obtained by this method are accurate.

In this paper, an approach of interactive contour delineation and automatic refinement is proposed. It allows users to quickly improve contours by a simple mouse action. A region which contains the interesting object is selected in the image, the program selects important control points from the region boundary, and Hermite cubic curves are used to fit the control points. Hence, the optimized curve can be revised by moving its control points interactively. It is the process of manual delineation and interactive revision. In order to improve the accuracy of contour delineation, the process of the curve refinement based on the maximum gradient magnitude is proposed. All the points on the curve are revised automatically towards the positions with maximum gradient magnitude. It is worthwhile to note that the use of curve refinement process makes it possible to locate contour delineation at the subpixel level. Meanwhile, the automatic curve refinement is extremely fast without using any iterations or optimizations, which might be time‐consuming or complicated for contour delineation process.

## II. MATERIALS AND METHODS

### A. The framework of contour delineation algorithm

**Figure 1:**
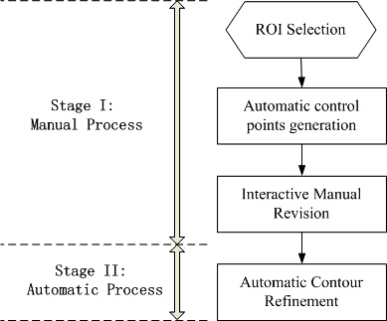
The framework of proposed contour delineation algorithm.

We explore digital curves to devise a contour delineation algorithm that consists of manual process and automatic process. As shown in Fig. 1, the proposed framework of contour delineation algorithm consists of two steps. First is the manual process that the user selects ROIs and manually revises automatically generated control points with mouse action. Second is the automatic process that the contour refinement is designed to locate the initial curve to be subpixel accurate. As pointed out in the Introduction, our proposed technique preserves the advantages of fully automatic techniques and clinical manual techniques. The manual process is extremely fast and efficient, which overcomes the drawbacks of former algorithms that are usually very time‐consuming and complicated. Meanwhile, skilled clinicians or doctors can also incorporate their valuable experiences in the process of manual revision to generate promising results. The stage of automatic contour refinement is devised to make the manually generated contour more accurate. In addition, it can also eliminate interobserver variation or bias. In other words, same results may be acquired from different skilled clinicians for the same ROIs.

### B. Hermite cubic curve and manual revision

#### B.1 Number of control points

Initially boundary of the region is generated by user's mouse motion, uniformly spaced control points will be generated automatically from the boundary. In general, number of control points should not be too large to make the manual process tedious because many control points are to be considered or revised by the user. Similarly, number of control points should not be small, when the shapes of ROIs are too complicated. In this work, we use a stationary value as the number of control points for each boundary of the region (e.g., 20 or 30 control points). The coordinates of control points will be defined by uniformly subdividing the initial region boundary by the stationary value. From our experiments, we set different stationary values according to the length of the boundary which can be calculated automatically after the user's mouse selection. For example, if the length of region boundary is smaller than 30 pixels, it means that the ROI is small and at least six control points are provided for manual revision to be accurate. The stationary value for such small ROIs should not be too small. Because few control points cannot generate considerable complicated shapes. The revision with few control points (e.g., less than five) cannot make the fitted curve enclose to the shape of small ROIs. On the other hand, the stationary value for small ROIs should not be too large. Large number of control points for small ROIs will make the adjustment tedious since control points are very close in small regions. If the length of region boundary is larger than 30 pixels but smaller than 100 pixels, ten control points are provided. Hence, if the length of region boundary is larger than 150 pixels but smaller than 200 pixels, 20 control points are presented. Finally, if the length of regions boundary is larger than 200 pixels, 30 control points are provided. Therefore, no more than 30 points are provided for large contours.

The advantage of our method for number of control points is to find a compromise between efficiency and accuracy. Frankly, more control points generated for manual revision will result in more accurate contour delineation, and the revised curve will accurately enclose to ROI. However, large control points for revision will be cumbersome, time‐consuming, and boring for users. Therefore, we set different stationary values according to the length of region boundary. It makes it possible to revise small contours with more control points to guarantee the accuracy. Meanwhile, it is efficient and fast to revise large contours with relatively small control points.

#### B.2 Hermite cubic curve

**Figure 2:**
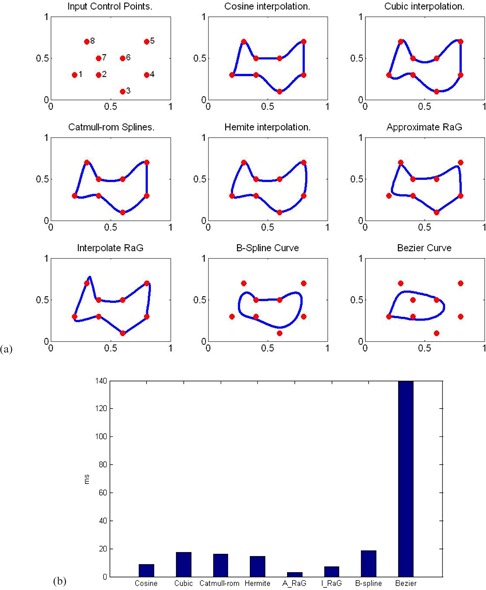
Comparison between Hermite cubic curves and other interpolation schemes.

Curve fitting methods are described that can accurately represent the region boundary with a parametric curve from above generated control points. Thus, the parametric curve generated by control points will replace the initial region boundary, generated by mouse click for further processing. Since the process of manual revision should be very fast and convenient for users, the selection of curve fitting methods is important. The main attributes are that they should be easy to compute and are stable. Actually, a number of interpolation or approximation methods have been proposed in the literature. Given eight control points in Fig. 2, we compare the characteristic of kinds of interpolation or approximation curves, such as cosine interpolation, cubic interpolation, and Catmull‐Rom spline and Hermite interpolation. Meanwhile, some classical parametric curves such as RaG, Bezier, and B‐spline are also compared. From our experiment, we find that interpolation scheme is better than approximate scheme for the manual revision, because it is more convenient for user to shift control points to the boundary of ROIs and automatically make the curve smooth to pass through those control points, rather than shifting control points to make the approximated curve enclose to the boundary of ROIs. Therefore, we prefer the interpolation curves to approximate curves for the manual revision.

As shown in Fig. 2, cosine interpolation, cubic interpolation, Catmull‐Rom spline, and Hermite interpolation exhibit very similar performance. The main contribute of these interpolation methods is that the estimated curve passes through all the given points. It clearly shows that Hermite interpolation achieves a higher degree of continuity and much smoother than other interpolation methods. Comparatively, smoother curves will make the parametric curve enclose real boundary of ROIs in images in terms of the round shape of the structures in radiation therapy. Although approximating RaG,^(32)^ Bezier or B‐spline curves are very smooth, they cannot pass through all the points. Interpolating RaG curve can pass through all the points, but it is not smooth in some regions, and some parts of the curve are stretched largely around some points. In addition, the consuming time of these methods are compared and shown in Fig. 2(b). Except for the Bezier curve, the time consumed by all other methods is shorter than 20 ms. The time required in generating the contour is much less than the response time of a normal user. Overall, the Hermite interpolating curve shows smooth characteristics while passing through all the points. Therefore, we use the Hermite curve on the interactive platform of contour delineation due to its simplicity, smoothness and interpolation.

Hermite cubic curve^(33)^ is a powerful tool to smoothly interpolate between key points. Given P0 and P1 represent the starting and ending points of the curve, and u0 and u1 represent tangent to how the curve leaves the start point and endpoint, respectively. Four Hermite basis functions are as follows:

(1) $$h1(s)=2s^{3}-3s^{2}+1$$

(2) $$h2(s)=-2s^{3}+3s^{2}$$

(3) $$h3(s)=s^{3}-2s^{2}+s$$

(4) $$h4(s)=s^{3}-s^{2}$$

These four vectors, P0, P1, u0, and u1, are simply multiplied with above four Hermite basis functions and added together. Then, the general form of Hermite curve is:

(5) P(s)=h1(s)P0+h2(s)P1+h3(s)u0+h4(s)ul

where scale 5 is to go from 0 to 1 with spacing Δs. In general, Δs is 0.1 in our experiments. That is, ten points will be generated for each segment between two control points, and the connection of these ten points will be the new digital curve that encloses the initial contour or boundary. In the process of curve refinements, we will show that these points generated with spacing Δs will determine the initial coordinates of curves for further refinement.

#### B.3 Manual revision

In our experiments of manual revision, all control points can be clicked on and dragged to altar the curves appearance. When one control point is selected for dragging, other control points will not react on dragging. Once the new revised position of the selected control point is determined by left mouse dragging, the new curve will be fitted again by Hermite cubic curve and displayed to replace the former boundary. The process of fitting Hermite cubic curve is real time with the movement of selected control points by mouse dragging. It is worthwhile to note that the process of manual revision is locally controlled, which means that the movement of a control points only affects the local area. This makes it more convenient and efficient than methods such as the linear interpolation or cubic interpolation, where the motion of a single control points affects the whole shape of the curve. Meanwhile, the complexity of manual revision can be controlled by setting the number of control point spacing which had been discussed.

**Figure 3:**
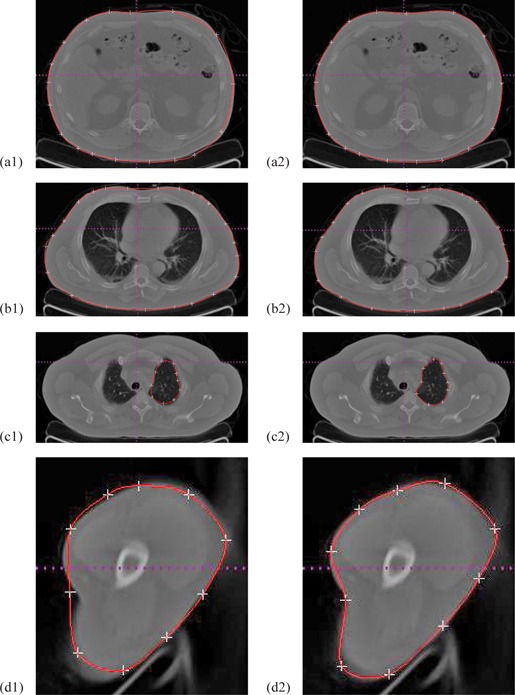
Initial drawing and interactive calibration results for four clinical CT images.

Figure 3 shows four cases of interactive calibration of real medical images using Hermite interpolation curves. The control points shown in white crosses are generated automatically from initial drawing. Selecting and shifting one control point locally will drag the red contour to satisfactory places until the red contour encloses the real boundary of interesting organs visually.

### C. The process of curve refinement

In general, the performance of manual delineation is highly dependent on user's experiences or visual inspection. At the given amount of time the manual revised delineation may be very accurate if a skilled clinician does it carefully. However, the revised delineation is prone to observer's error or bias. Different clinicians may manually delineate slightly different contours, and it might complicate the following quantitative measurements to monitor disease progression. Therefore, accurate contour delineation of ROIs is essential for postprocessing in clinical applications. In this work, the process of curve refinement is fully automatic. Once the manual delineation and revision is done, the process of curve refinement will be carried out automatically.

The purpose of curve refinement is shifting the points on manual revised curve to new positions with maximum gradient magnitude in local areas using gradient information in images. Our proposed curve refinement is simple, but very efficient and accurate. Given the curve and coordinates of points on the curve, the process of curve refinement is as follows:

Step 1. Compute the gradient information of the original image by Gaussian derivative filters, G. This is achieved by filtering the image with 1D operators that are computed using the following formula:

(6) $$G(x)=\frac{1}{\sqrt{2\pi \sigma }}e^{-\frac{x^{2}}{2\sigma^{2}}},\frac{\delta G}{\delta x}=-\frac{x}{\sigma^{2}}G(x)$$

where σ is the standard deviation of the Gaussian function. In the work, σ should be large for low‐resolution images, and vice versa.

Step 2. Select one point Pi and another two points — Pi−1,Pi+1 — at the left and right of point Pi on manual revised Hermite cubit curve, respectively.

Step 3. Calculate the normal direction, which is also the direction of the searching space, from point Pi−1 and Pi+1 for the point Pi.

Step 4. Set the threshold distance of the step length as d=1 to 5 pixels, and search along the direction of the normal within ±d to find out the position with the maximum gradient magnitude (x_d, y_d). Here, bilinear interpolation is used to achieve subpixel localization precision.

Step 5. Shift the point Pi to the new position (x_d, y_d) and generate the final curve from points with new positions.

**Figure 4:**
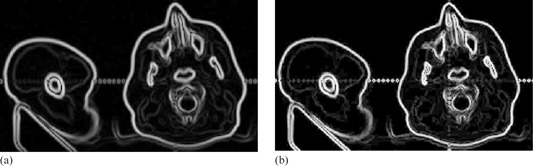
Gradient magnitudes (a) using the derivative of the Gaussian with 1.0, and (b) using derivatives directly.

In Step 1, the main advantage of calculating gradient using the derivative of the Gaussian is that the Gaussian has a smoothing effect and the scale parameter controls the amount of noise reduction. After calculating the partial derivatives of Gaussian, weak edges response or noise in images is eliminated by applying a nonmaximal suppression procedure for the gradient information. As shown in Fig. 4, the result of calculating gradient magnitudes using the derivative of Gaussian is compared with gradient magnitudes using derivatives. It clearly shows that the gradient information generated by the derivative of the Gaussian is more distinctive on boundaries than the result using derivatives directly. Distinctive magnitudes of boundaries are beneficial for further curve refinement. We expect to automatically revise the points on the manual curve to the positions of locally largest gradient magnitudes along certain directions.

**Figure 5:**
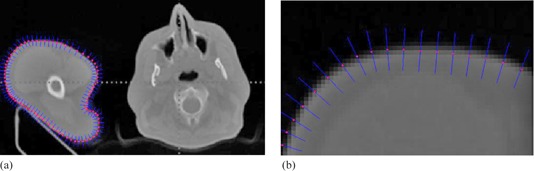
Refined direction points (a) on initial manual revised curve; (b) enlarged image.

Steps 2 and 3 define the direction that the point on manual curve should be automatically refined. Since the point Pi and its adjacent points Pi−1 and Pi+1 are very close to each other at the manual curve, the tangent direction at point Pi is calculated directly by fitting a line of three points, and normal direction which is also the automatically refined direction is perpendicular to the tangent direction. Similarly, each point on manual curve is automatically refined in this manner. Figure 5 shows the case of direction calculated by its adjacent points. Red dot shows the position of points on the manual revised curve, and the blue segment shows the direction of refinement at one point, which is calculated by the point and its adjacent points. Figure 5(b) shows an enlarged region in Fig. 5(a).

**Figure 6:**
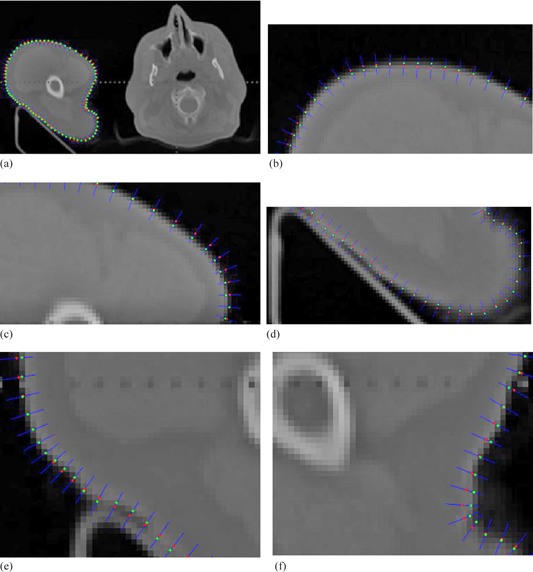
A case of curve refinement.

Step 4 restricts the distance threshold of the searching space along the normal direction. Usually, the direction of the searching should be on two sides of the point. Once the gradient value at the new position is largest among the search space for this point, the new position will be the final refined position for the point in Step 5. It should be noted that the search spacing Ad can be very small (e.g., Δd=0.1pixel). If the searching distance threshold d is two pixels, the searching space will be 21 units. In order to generate gray values for each unit among the search space, bilinear interpolation is used to achieve subpixel localization precision. Figure 6 shows a case of curve refinement. Red dot shows the point on manual revised curve, and green dot shows the point that had been refined in Fig. 6(a). To be observed more clearly, Fig. 6(b‐f) show enlarged regions in Fig. 6(a). The refined positions appear to be more natural and accurate than original positions.

**Figure 7:**
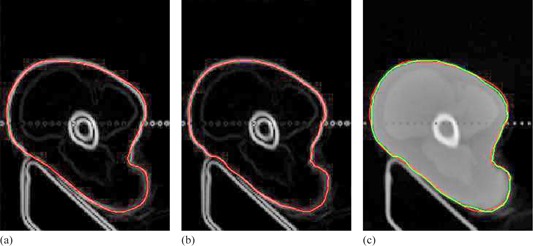
Manual revised contour (a); (b) automatic refinement curve; (c) fusion of the two delineations in the original image.

Meanwhile, Figs. 7(a) and (b) show the comparison results of delineation before and after the process of curve refinement in gradient magnitude images. Figure 7(c) shows the manual revised delineation and automatic refined delineation together in the original image, where red curve shows the manually calibrated contour and the green curve shows the automatic curve refinement of the contour. It is clear that the curve refinement improves the manually calibrated result. Overall, the automatic curve refinement plays an important role in accurate contour delineation.

**Figure 8:**
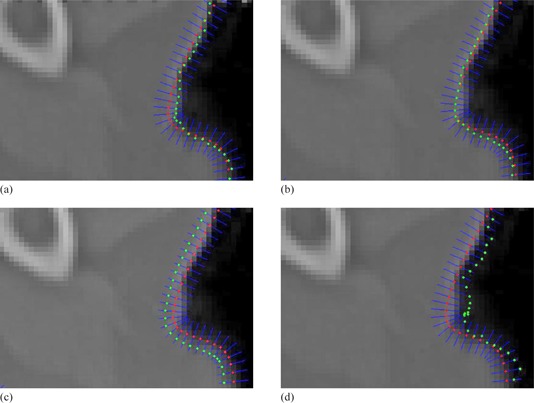
The localization of refined points on boundary with different values of Δd.

There are two parameters in the process of curve refinement: d and Δd. Since the initial curve has been revised manually, it is assumed the initial curve is very close to the real boundary. Therefore, it is generally adequate that the distance threshold value d is 2 or 3 pixels. In addition, the search spacing Δd is usually 0.1 pixel in our case. With the utilization of bilinear interpolation, the localization of boundaries can be subpixel rather than pixel's accurate level. However, in our experiments even smaller Δd will not result in more accurate localization of boundaries due to the interpolation errors or image noise. Figure 8 shows the localization of refined boundaries with different values of Δd in our experiments. The performance of the refinement appears to be best when the search spacing Δd is 0.1 pixel. Larger values of Δd will not result in accurate positions with maximum gradient magnitudes. Similarly, too small values of Δd generate unreliable results due to interpolation error or image noise, as shown in Fig. 8(d).

**Figure 9:**
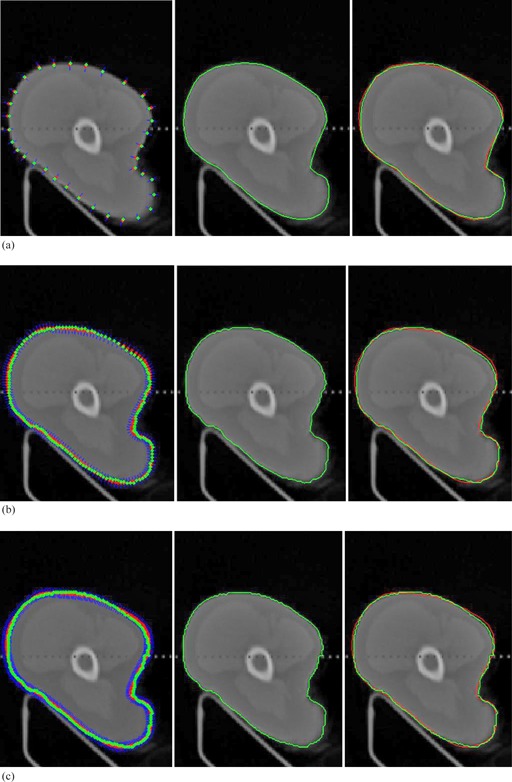
Simulation results of contour delineation with different spacing Δs.

In addition, the number of points on manual revised curve is determined by spacing Δs when scale s goes from 0 to 1, and is essential to the accuracy of final delineation. Intuitively, more points on manual revised curve will result in more points for further refinement, and the final delineation is prone to be more accurate. Figure 9 shows our simulation results of contour delineation with different spacing Δs. From the results, we analyze that with the decrease of spacing Δs the final contour refinement provides detailed information. Therefore, small spacing value Δs in parametric curves makes it possible to locate accurate boundaries. Generally, it is enough to generate accurate boundaries when spacing Δs is 0.1 in our experiments. Occasionally, if the shape of organs or ROIs in medical images is complicated, it is necessary to set relatively smaller spacing value of Δs.

Overall, our proposed contour delineation consists of two steps: manual control point revision and automatic refinement. The process of manual control point revision is coarsely reducing boundary errors of manual drawing, and the process of automatic refinement is then finely generating accuracy boundaries. In clutter areas, the margin of errors allowed for contour refinement should be small to reduce the influences of noise or unrelated edges. In such cases, the manual revised process should make the initial drawing approximate to the true boundary by control point revision. The precondition of automatic contour refinement is that the margin of errors is not too large after the process of manual control point revision. In the process of contour refinement, the margin of errors can be allowed is usually 3‐5 pixels in our proposed implementation on the platform, and the margin of errors is determined by a predefined distance threshold, d. Larger d will show larger searching space to refinement process along the normal direction, and vice versa. If the margin of error in the process of manual revision is larger than the distance threshold, d, the process of contour refinement will become failure in the proposed method. Therefore, the process of manual revision should guarantee that the margin of error is smaller than the distance threshold, d.

## III. RESULTS

The proposed interactive tool can be used to segment ROIs or delineate contours in images. We first tested the performance of the proposed interactive tool on synthetic images in order to make sure that the ground truths of the boundaries were known. We also tested the performance of the proposed method from kinds of medical images, such as CT images, MRI images, and ultrasound images in clinical applications. Finally, we tested running times for the proposed method. In addition, classical contour model, such as Snake, is used for the comparison. To make sure that the Snake model can generate best results, optimal parameters are selected according to Kass and Witkin^(7)^ and the initial contour of Snake is manual drawn and close to the final solution.

### A. Contour delineation in synthetic images

**Figure 10:**
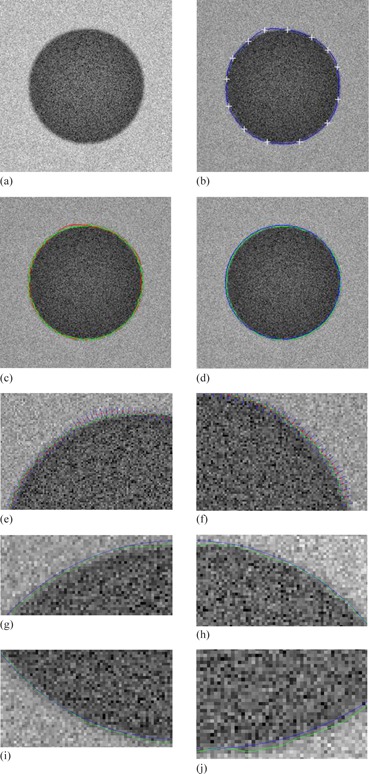
Contour delineation by the proposed method: (a) original image; (b) manual revision with control points; (c) the manual revised contour (red) and the curve refined contour (green); (d) blue shows the ground truth of the boundary, and green shows the curve refined contour by the proposed method; (e‐f) show parts of curve refinement; (g‐j) show parts of enlarged area in (d).

**Figure 11:**
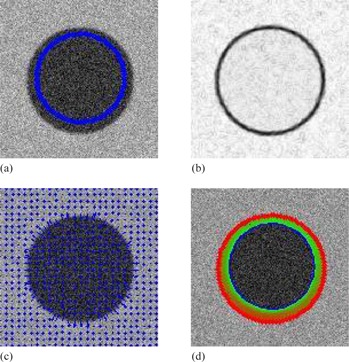
Contour delineation by the basic Snake model for the synthetic image: (a) original image and initial contour; (b) the external energy; (c) the external force field; (d) Snake movement. Red contour shows the final delineation by the Snake model.

The proposed interactive tool is firstly implemented on synthetic images. Note that the synthetic image is generated from the original binary image by corrupting it with additive Gaussian noise. Thus, the ground level truth of the boundary is known. As shown in Fig. 10, a black circle with radius 85 pixels is generated in the center of an image of size 256×256 pixels and the intuitive results are achieved by the proposed method. Figure 10 (g‐j) shows parts of enlarged area in Fig. 10(d). It can be clearly observed that the boundary delineation obtained by the proposed method is visually good and very close to the ground level truth boundary in the noisy synthetic image. The Snake model can delineate correct boundary in such cases where the initial contour is a bit away from the actual boundary, as shown in Fig. 11. We can find that both the proposed method and Snake model can obtain promising results for the synthetic image.

### B. Clinical medical images

The delineation results in medical images using proposed method are presented, and the results of initial manual drawing, manual revision, and curve refinement are all shown, respectively. Additionally, the basis Snake model is also used for the comparison. A formal quantitative evaluation of the proposed method for real medical images is not contained in the paper due to the interactive nature of the method and the unknown ground truth data.

#### B.1 CT images

**Figure 12:**
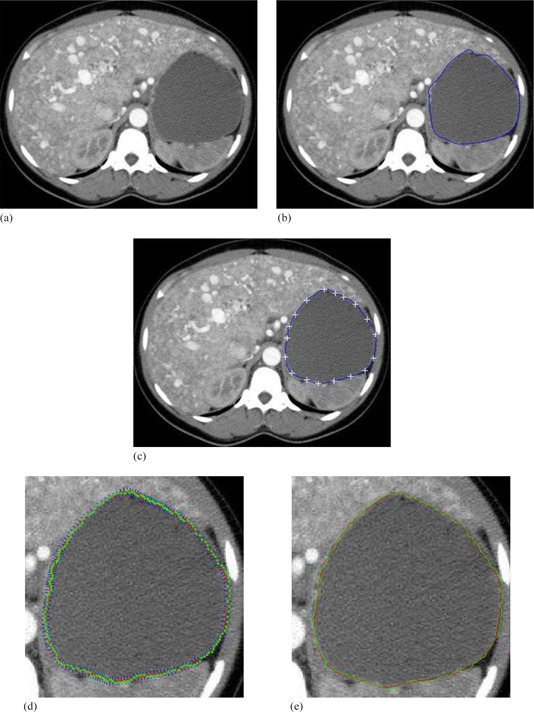
Contrast‐enhanced axial CT scans through liver:^(34)^ (a) original image; (b) initial manual delineation; (c) manual revision with control points; (d) curve refinement; (e) the manual revised contour (red) and the curve refined contour (green).

The CT image in Fig. 12 is obtained from Learning Radiation website,^(34)^ and shows the contrast‐enhanced axial CT scans through liver.

#### B.2 MRI images

**Figure 13:**
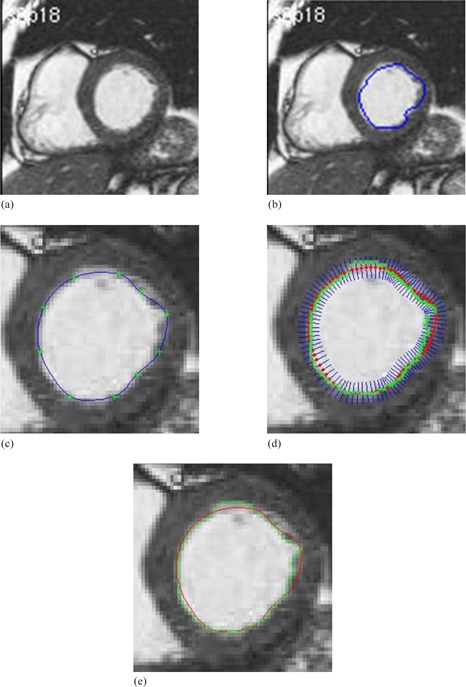
Left ventricle in cardiac from MRI images:^(35)^ (a) original image; (b) initial manual drawing; (c) control point generation and manual revision; (d) automatic curve refinement; (e) red shows the manual revision and green shows the final refined delineation.

Figure 13 shows the left ventricle in cardiac from MRI images of the heart. Development of contour detection techniques for the left ventricle is required to be able to reduce the total analysis time and to reduce the inter‐ and intraobserver variability associated with manual contour tracing.

#### B.3 Ultrasound images

**Figure 14:**
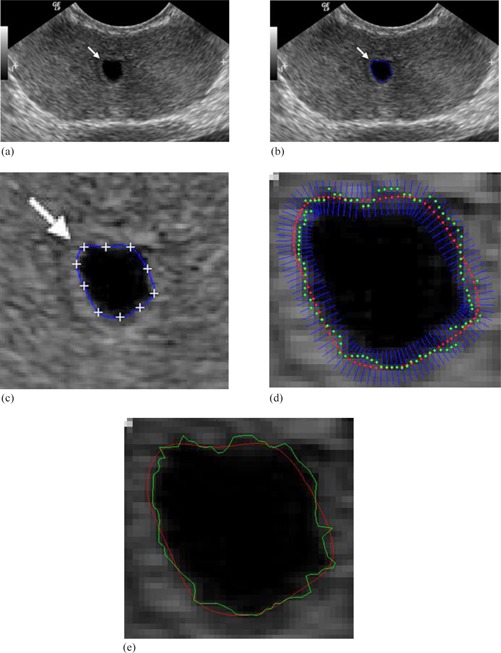
Transrectal ultrasound image of the prostate:^(36)^ (a) original image; (b) initial manual drawing; (c) control point generation and manual revision; (d) automatic curve refinement; (e) red shows the manual revision and green shows the final refined delineation.

Figure 14 shows the contour delineation result of a transverse image of the prostate in a young male that demonstrates a small midline cystic structure (arrow) represent a utricle cyst. Due to low resolution and low contrast of ultrasound images in addition to the speckle noise, either manual delineation methods or fully automatic delineation methods often contain errors for contour delineation. The green contour shown in Fig. 14(e) displays the final result of contour delineation by the proposed method, which appears to be accurate visually.

**Figure 15:**
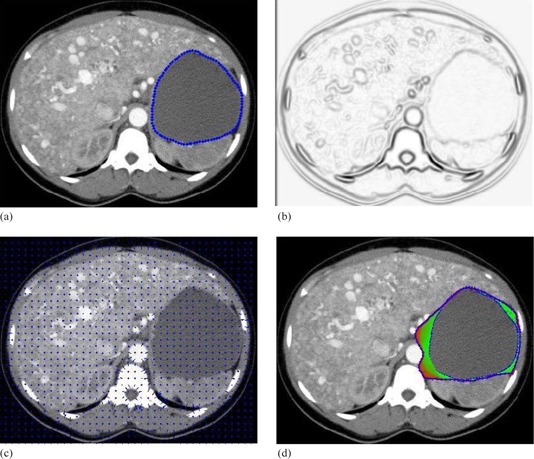
Contour delineation by the basic Snake model for the contrast‐enhanced axial CT image: (a) original image and initial contour; (b) the external energy; (c) the external force field; (d) Snake movement. Red contour shows the final delineation by the Snake model.

**Figure 16:**
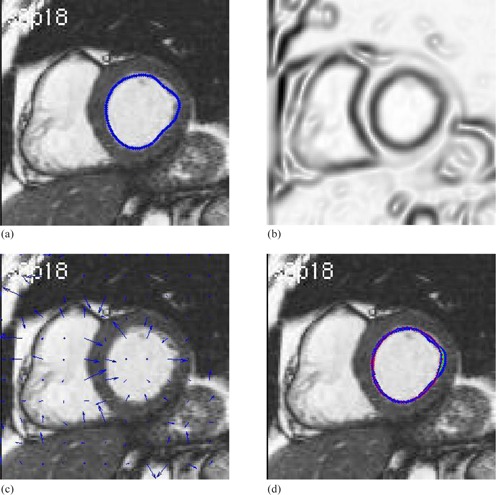
Contour delineation by the basic Snake model for the MR image of left ventricle in cardiac: (a) original image and initial contour; (b) the external energy; (c) the external force field; (d) Snake movement. Red contour shows the final delineation by the Snake model.

**Figure 17:**
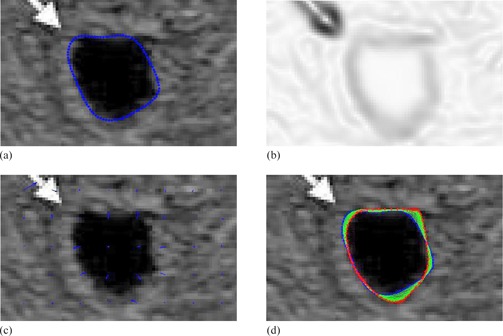
Contour delineation by the basic Snake model for the ultrasound image of the prostate: (a) original image and initial contour; (b) the external energy; (c) the external force field; (d) Snake movement. Red contour shows the final delineation by the Snake model.

**Figure 18:**
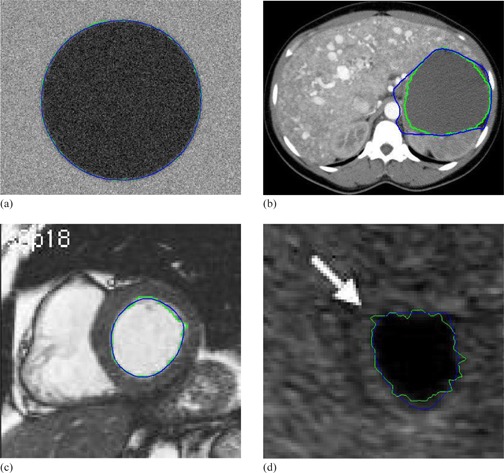
Contour delineation by the basic Snake model and proposed method with the same initial contour that is close to the true boundary: (a) synthetic; (b) CT; (c) magnetic resonance (d) ultrasound. Blue shows the final contour generated by the basis Snake model, and Green shows the final contour generated by the proposed method.

The results of the proposed method for medical images as shown in Figs. 12, 13, and 14 are visually good and promising. They demonstrate that the proposed method yields the refined contours that are very close to the visual inspection or perceptual observations. To fairly compare the proposed method with Snake model, same start contours are drawn for both methods, and the start contours are all very close to the true boundaries. However, the results from the well known Snake model are not user satisfactory for these cases as shown in Figs. 15, 16, and 17. The final contour obtained by the proposed method and the Snake model are shown in Fig. 18, and the time consumed by both methods are also compared and shown in Table 1. In Fig. 18, the results obtained by our proposed method appear to be much better than Snake model. Although final contours obtained from Snake model are much smoother, they are often away from the true boundaries. The reason is that the sharp edge is smoothed out by the Snake's internal energy which resists high curvature. For example in CT image, the boundary of the object is not salient and the result of Snake model contains large errors. Comparatively, the results obtained from the refinement of the proposed method are robust and no obvious errors in final contours can be observed. Moreover, the time taken by Snake model is much larger than the process of refinement in our proposed method. The fact is that we apply simple local search to generate the final contour, and it will make contour delineation simple and fast for clinical applications.

In general, Snake model works well where there are clearly defined boundaries in such medical images and the shape of object is reasonably smooth, since sharp edges will be smoothed out by the Snake's internal energy, which often resists high curvature. Once the image is in low resolution and the true boundary is not distinctive, such as above cases of clinical CT and ultrasound images, the final contour of Snake model will be wrong. Thus, the Snake model is not robust in clinical applications. Moreover, Snake model is often time consuming when the start contour is not very close to the final solution and needs tenth of seconds or more for iteration optimization. For those reasons we develop a fast, efficient, and robust contour delineation approach for clinical applications.

Generally, the initial contour for Snakes is often required close to the final solution. If the Snake is initialized “too far” from the object boundary, it is possible that the contour may not be able to converge onto object boundary. Actually, the process of curve refinement in the proposed method has the similar problem. If the initial contour is far away from the actual contour, the process of curve refinement may fail when the search space is limited to 5 to 10 pixels, which already implies the initial contour should be close to the actual contour. In order to make the process of curve refinement work properly, the process of manual revision in the proposed method by revising control points can make the initial contour close to the actual contour. If the same initial contour is applied and replaced the contour refinement with Snake method interactively, the Snake method may produce fine result, as well. However, the key contribution of the process of curve refinement in our proposed method is its simplicity and can be implemented relatively easily. As has been pointed out, the time required of Snake model is much longer than the process of curve refinement in our proposed method with same initial contours as shown in Table 1. In addition, the limited search space in the proposed method can also restrict the displacement of final contours from the close initial contour, and it can often avoid oversegmentation of objects with blurring edges. For example, the comparison results of the CT image are shown in Fig. 12 and Fig. 15; the Snake method oversegments the object, but the proposed method works well.

Our interactive tool provides means to generate satisfactory contour delineation for clinicians, whether the clinician is a skilled expert or a beginner. Compared with fully automatic delineation methods, the proposed interactive tool incorporates user's operation and makes it more robust to complicated situations in images, which seems to be useful and convenient for real clinical applications. Compared with manual delineation, the described process provides highly efficient means of manual revision, as well as automatic curve refinement to generate high‐accurate boundaries.

**Table 1:** The comparison of time taken between Snake model and proposed method with the same initial contour that is close to the true boundary (sec)

	*synthetic*	*CT*	*Magnetic Resonance*	*Ultrasound*
Snake Model	10.68	9.52	6.46	6.76
Proposed Method	0.20	0.24	0.16	0.15

## IV. DISCUSSION

The described interactive tool has been used to segment 2D medical images. Contour delineation of 2D images of size 512×512 pixels may take a few seconds. The time includes the initial manual drawing step, the interactive manual revision step, and the automatic refinement process on a laptop with CPU 3.3 GHz and 4G RAM. The Hermite curve fitting in interactive manual revision step is real time with mouse action and takes no more than 0.02 seconds for each fitting due to the high‐efficiency of Hermite cubic curves. The curve refinement process takes no more than 0.3 seconds, even for large contours with more than 500 points as shown in test results in Table 1. Therefore, the time taken for curve fitting and curve refinement is very small, and can be almost ignored in the whole process in clinical applications.

It should be noted that the whole process for contour delineation is real time for users with mouse actions, whether for large ROIs or smaller ROIs in our experiments. Meanwhile, the process of automatic curve refinement is extremely fast to locate high‐accurate boundaries due to its simple implementation. Usually, it may take several seconds to interactively revise a result which is fully determined by users’ experience. The interactive manual revision process can continue until a coarse result (may not be, but can be, satisfactory) is achieved from user's inspection. Therefore, more running time is spent to initially drawing contour of a ROI and manually interactive revision of associated control points, which is determined by clinicians and size of ROIs. Generally, large ROIs may take more time for users to draw an initial contour, and more time is needed to manually revise various control points until a coarse result is achieved.

If the boundary is distinctive in images, the process of curve refinement will play an important role for accurate boundary delineation, and our proposed method can obtain good results. In the case of noisy boundaries, the process of manual revision will contribute more to the final accuracy, and the distance of refinement needs to be small in order to decrease the influence of noise. The combination of manual revision and automatic refinement makes our method efficient, accurate, and robust in noisy images. Therefore, both the manual revision and refinement are important for contour delineation in our proposed method.

It can also be an issue if autorefinement results are not satisfactory in our proposed method. This case is possible in clinical applications if the boundary of ROIs is blurred or too noisy. On our software platform, we present remedy for this drawback in the following way. If the final refined boundary contains some errors, we can treat this boundary as an initial manual drawing and control points will be generated on this boundary. We can manually revise the boundary by mouse dragging control points. In this way, the errors of automatic contour refinement can be reduced or eliminated manually. On our software design, the process of interactive manual revision and the process of automatic contour refinement are implemented independently. If the final contour refinement is not satisfactory, the final contour can be reselected by the mouse action (double left mouse click) again as the initial contour for further manual revision.

Moreover, it is worthwhile to note that generating uniformly space control points in the proposed method does not take into account the curvature of the boundary, and this may definitely affect the accuracy of the contour delineation in the process of manual revision. Typically, more control points should be generated for the region of boundary with large curvature, and vice versa. However, the purpose of using equal distance and limited number of control points in our proposed method is to make the process of manual revision simple and efficient. It is true that limited uniformly spaced points may create problem for extreme case, such as a narrow long structure or very complicated shapes. Actually, more control points for manual revision may be better, but it will be tedious for users’ adjustment. Practically, the initial manual revision of control points is to reduce large errors of initial manual drawing, and obvious errors can be reduced by shifting several related control points.

## V. CONCLUSIONS

Purely manual contour delineation may be very accurate, but it is time‐consuming and tedious for users, and fully automatic delineation often involves errors and is sensitive to noise in images. The idea of this work has been to develop an interactive tool that can make the manual process highly efficient, and it enables a user to revise delineation with few selected control points by the manual process. Meanwhile, the process of curve refinement makes it possible to locate contour delineation to be subpixel accuracy, which can eliminate observer variation or bias of manual delineation. The extension of this idea to 3D case is straightforward. We will generate control points on digital surfaces obtained from coarse thresholding segmentation in 3D for manual revision and automatic refinement. Since the purpose and application of our research are treatment planning of IGRT, this technique is directly applicable to the target delineation in IGRT. We believe that our proposed technique can be extended to general medical images in broad applications, and is applicable to various kinds of clinical applications. Our future work will also explore more sophisticated shapes in images for contour delineation.

## ACKNOWLEDGMENTS

This work is supported by the grant from China Postdoctoral Science Foundation (2013M530740), and in part by grants from National Natural Science Foundation of China (NSFC: 81171402), NSFC Joint Research Fund for Overseas Research Chinese, Hong Kong and Macao Young Scholars (30928030), National Basic Research Program 973 (2010CB732606) from Ministry of Science and Technology of China, and Guangdong Innovative Research Team Program (No. 2011S013) of China.

## Supporting information

Supplementary Material
