# Supplementary material for: Interactive contour delineation and refinement in treatment planning of image‐guided radiation therapy
Source: J Appl Clin Med Phys. 2014 Jan 6;15(1):141–66. doi: 10.1120/jacmp.v15i1.4499 (PMC5711244; doi:10.1120/jacmp.v15i1.4499)
Supplement: Supplementary file 1 — Supplementary Material [file ACM2-15-141-s001.docx]

**Interactive contour delineation and refinement in treatment planning of image guided radiation therapy**

Wu Zhou

Ph.D and Assistant Professor

Key Laboratory for Health Informatics, Shenzhen Institutes of Advanced Technology,

Chinese Academy of Sciences, Shenzhen 518055

Phone: 86-13711784840; 86-75586392295;

Fax: 86-75586392299

Email: [wu.zhouo@siat.ac.cn](mailto:wu.zhouo@siat.ac.cn); [zhouwu787@gmail.com](mailto:zhouwu787@gmail.com)

Yaoqin Xie

Ph.D and Full Professor

Key Laboratory for Health Informatics, Shenzhen Institutes of Advanced Technology,

Chinese Academy of Sciences, Shenzhen 518055

Phone: 86-18923712562; 86-75586392281;

Fax: 86-75586392299

Email: [yq.xie@siat.ac.cn](mailto:yq.xie@siat.ac.cn);
